# A proof of concept for improving comparability of dosimetry audits through centralised planning

**DOI:** 10.1016/j.phro.2025.100879

**Published:** 2025-11-29

**Authors:** José Antonio Baeza-Ortega, Lauren May, Mohammad Hussein, Sarah Porter, Alisha Moore, Peter B. Greer, Catharine H. Clark, Joerg Lehmann

**Affiliations:** a School of Information and Physical Sciences, University of Newcastle, Newcastle, Australia; b Radiotherapy and Radiation Dosimetry, National Physical Laboratory, Teddington, United Kingdom; c Radiation Therapy Quality Assurance, Trans-Tasman Radiation Oncology Group (TROG Cancer Research), Newcastle, Australia; d Department of Radiation Oncology, Calvary Mater Hospital, Newcastle, Australia; e Department of Radiotherapy Physics, University College London Hospital, London, United Kingdom; f Department of Medical Physics and Bioengineering, University College London, London, United Kingdom; g Institute of Medical Physics, University of Sydney, Sydney, Australia

**Keywords:** Quality assurance, Sensitivity, Patient specific quality assurance, SEAFARER

## Abstract

**Background and purpose:**

The role of dosimetry audits is well established in the development and verification of radiotherapy safety. Differences in planning and beam modelling make inter-centre comparisons challenging, which can be addressed through distribution of centrally created plans. This study developed a centralised planning approach applicable to multiple audit methodologies, using an example of remote patient specific quality assurance assessment, increasing the interpretability of results and facilitating automation and scalability.

**Material and methods:**

Starting with an established plan which met all clinical goals, a commercial dose mimicking algorithm was used to replicate this plan to be suitable for multiple treatment machines. Beam and machine limitation data were collected from participating centres to develop universally acceptable beam models. The influence of variation in beam modelling parameters among centres was assessed by creating additional models using the 2.5th, 25th, 75th and 97.5th percentiles of previously reported data. Multi-leaf collimator angle and leaf position, gantry angle and output deviations were then introduced into copies of these plans.

**Results:**

Introduced delivery errors caused consistent change in dose metrics across machine models (excluding outliers) with a median (range) standard deviation of 1.0 % (from 0.1 % to 1.7 %) demonstrating similar robustness. Beam model variation did not change whether simulated delivery errors were clinically impactful or not for 95 % of tested plans.

**Conclusion:**

This study lays the foundation for future standardised methodology for dosimetry audits by providing a centralised planning approach that allows a more consistent assessment of centres.

## Highlights

• A centralised planning approach to auditing methodologies was developed.
• This work lays the foundation for future patient specific quality assurance audits.
• Beam model variation did not significantly affect impact of errors in 95 % of plans.
• Plans of similar quality and robustness were developed for several accelerator models.

## Graphical abstract

**Figure:**
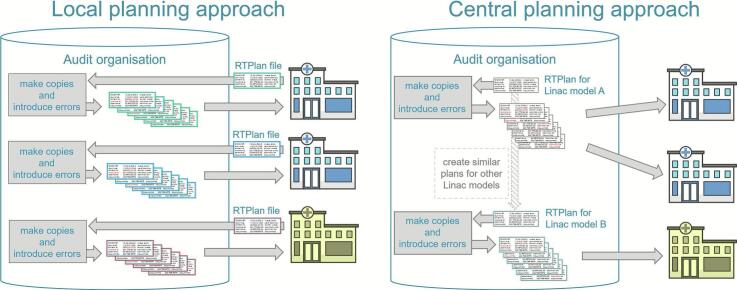

## 1 Introduction

The success of radiotherapy, including patient survival, has been shown to be impacted by its quality [1]. Radiotherapy dosimetry audits have been well established as a way of assessing and identifying issues with treatment delivery and safety [2]. However, differences in planning techniques, equipment and beam modelling between centres can make inter-centre comparisons in audit performance more difficult, for example, when comparing individual centres’ ability to catch errors via their patient specific quality assurance (PSQA) systems.

Previously, a novel approach was introduced to assess the ability of a radiotherapy centre’s clinical PSQA methodology to detect delivery errors through purposely introduced treatment delivery errors [3]. Participating centres submitted a treatment plan file created locally based on a provided data set and instructions. Copies of the provided plan were made and treatment delivery errors were introduced into them. Centres performed their clinical PSQA method and reported which plans passed or failed. The results highlighted the limited ability of some PSQA methods to detect clinically relevant errors but also showed that locally created plans differed greatly in their robustness towards the introduced errors [3].

For PSQA audit results to be comparable across centres in multi-institutional studies, plans with similar changes in dose as a result of modifications need to be delivered. These should be a balanced mix of plans that “should pass” and “should fail” PSQA. Distribution of a centrally created set of plans, in addition to offering consistent plan quality and robustness, also reduces the workload for the centres and simplifies the creation of plans with delivery errors making the study more easily scalable. Ideally, the same set of plans should be used for all centres, however this is not possible with a variety of machine and multi-leaf collimator (MLC) models. Additional factors need to be considered including catering to machine limit constraints which vary among centres and consideration of expected dosimetric differences due to beam modelling variation.

This work aimed to develop a centralised planning approach applicable for use in dosimetry auditing, by creating dose-equivalent treatment plans for multiple linac-MLC combinations and introducing a set of delivery errors to those plans.

## 2 Materials and methods

### 2.1 Machine limits and beam model parameters

To consider differences in user-adjustable machine limits and beam modelling parameters, relevant data were collected from participating radiotherapy centres using REDCap electronic data capture tools hosted at Hunter Medical Research Institute, Australia [4,5]. These data included participant system information, machine limits, beam modelling parameters, PSQA systems and treatment sites of interest.

Using the RayStation treatment planning system (TPS) (2023B v.14, RaySearch Laboratories, Stockholm, Sweden), 6 MV photon beam models were created for the following treatment machines and MLC configurations: Elekta Versa HD with Agility MLC (VHD), Varian TrueBeam with the Millenium MLC (TBM), Varian TrueBeam with HD120^TM^ MLC (TBHD), and Varian Clinac with Millenium MLC (CM) along with a 6FFF beam for the Varian Halcyon (HY). Beam modelling parameters were set to match corresponding median values in the Imaging and Radiation Oncology Core (IROC) Houston survey data [6]. As there was no IROC data for the TBHD, a locally available beam model was used.

To ensure deliverability, created plans must not exceed the machine limits used by the participating centres. Survey data from participants were analysed to determine appropriate machine limits to accommodate the majority of centres. As very strict machine limits restrict the ability to optimize complex plans, an additional beam model was created for centres with outlying values.

Slight differences in dose distribution will exist due to small variations in machines and therefore corresponding beam models across participating centres. In order to assess the effect of variation in beam model parameters across centres, additional beam models were created for two of the machines: TBM and VHD. Eight fluence modelling parameters available in the RayStation TPS were used: effective source width (in the X and Y directions), MLC transmission, tongue and groove width, leaf tip width and three parameters modelling the effective position of the rounded MLC leaves. Each of these parameters were determined to be in general either proportional or inversely proportional to an increase in calculated dose. Beam models were then created using the IROC survey data [6] for 2.5th, 25th, 75th and 97.5th percentiles, so that the 2.5th percentile model calculates ‘colder’ and the 97.5th percentile calculates ‘hotter’. Therefore, parameters which are inversely proportional to increased dose (primary source X and Y width; and tongue and groove) were selected from the opposing percentile accordingly.

### 2.2 Treatment planning

For use in demonstrating the proposed methodology, a head and neck volumetric modulated arc therapy (VMAT) treatment plan for a patient geometry consisting of two targets with a prescription of 65 Gy to PTV1 (volume = 430 cm^3^) and 54 Gy to PTV2 (volume = 341 cm^3^) delivered in 30 fractions was created by an expert planner for a VHD. Optimization objectives are outlined in Table S1, supplementary material. The Fallback planning module was used to mimic the mother plan dose for the other machine and MLC models. This module uses the mother plan’s dose distribution, independent from the plan, to produce a similar dose distribution for a new plan with user specified characteristics (e.g. beams). Optimisation is based on both voxelwise comparison and dose volume histogram (DVH) comparison. The HY was excluded in this case due to the absence of a 6 MV flattened field beam, however the same method would be applicable in other circumstances. The original optimisation parameters were then used to reoptimize each of the fallback plans (while still maintaining the “mimic dose” objective) until each of them complied (within ±1 %) with the clinical goals (Table S2, supplementary material) based on the ART DECO trial guidelines [7]. These fallback plans will be referred to as baseline plans.

Complexity of baseline plans was assessed using a range of previously defined complexity metrics: median MLC gap, small aperture score (SAS10), mean MLC speed, plan irregularity, plan modulation, modulation complexity score, mean gantry speed and gantry speed modulation. These were calculated using PlanAnalyzer (v.11) [8] implemented in MATLAB (Mathworks, Massachusetts, USA).

### 2.3 Simulated treatment delivery errors

For each of the baseline plans, ten copies containing delivery errors either alone or in various combinations were created via the inbuilt scripting module of RayStation using IronPython (v.3.4). These errors included modifications of: MLC bank positions (contracting or retracting), collimator angle rotations, machine dose outputs (in the form of monitor unit (MU) scaling) and gantry angle rotations (constant angle variation for the entire arc) (Table 1). Several machine QA tests exist with the potential to detect these errors with varied recommended test frequency [9]. Collimator and gantry angle indicators are recommended to be tested monthly; X-ray output constancy is recommended to be tested daily/weekly and MLC leaf positions have a variety of tests recommended weekly, monthly and annually. However, the magnitudes of these errors were selected to be less than the tolerance accepted by routine machine QA [9].

**Table 1:** Modifications included in each plan.

Plan	Introduced errors
1	Collimator – 0.8°, gantry − 0.8°
2	Collimator – 0.8°, gantry + 0.8°
3	MLC banks retract 0.4 mm
4	MLC banks contract 0.4 mm
5	MLC banks retract 1.0 mm
6	MLC banks contract 1.0 mm
7	MLC banks retract 1.0 mm, MU + 1.9 %, gantry – 0.8°
8	MLC banks retract 1.0 mm, MU + 1.9 %, collimator + 0.8°
9	MLC banks contract 1.0 mm, MU − 1.9 %, collimator − 0.8°
10	MLC banks contract 1.0 mm, MU − 1.9 %, gantry + 0.8°

The impact of plan modifications was assessed by recalculation of the dose in the TPS with comparison back to the baseline plan dose distribution. Plan modifications were deemed to have a clinically significant dosimetric effect if the maximum dose to 95 % of the volume (D_95_) of either PTV1 or PTV2 was reduced by >5 % or if the maximum dose, defined here as dose covering 0.03 cm^3^ (D_0.03cc_), was increased by >5 % for either the brainstem or spinal cord.

## 3 Results

The most restrictive machine limit values from the survey data for each machine and MLC model are shown in Table 2. Two departments had outlying values for the VHD minimum dynamic tip gap and were excluded from the ‘standard’ VHD data. An additional ‘secondary’ VHD dataset is also shown which includes these outlying values. Using beam models informed by this data, similar DVH parameters of the baseline plans were achieved (Table 3).

**Table 2:** Most restrictive machine limits used by surveyed departments for each machine and MLC model.

Parameter	TBM/TBHD	VHD	VHD secondary	HY
Gantry angle start (°)	181	181	181	181
Gantry angle stop (°)	179	179	179	179
Maximum jaw speed (cm/s)	2.0	3.0	3.0	N/A
Maximum leaf speed (cm/s)	2.0	3.0	3.0	5.0
Minimum static leaf tip gap (cm)	0.5	0.5	0.5	0.0
Minimum dynamic leaf tip gap (cm)	0.50	0.50	1.00	0.05
Minimum static arc dose rate (MU/min)	100	150	150	27
Minimum dynamic arc dose rate (MU/min)	100	150	150	800
Minimum gantry angle speed (°/s)	0.5	0.1	0.1	0.0
Maximum gantry angle speed (°/s)	4.8	5.0	5.0	12.0
Gantry degree per control point (°)	2.0	2.0	2.0	2.0
Minimum MU per gantry degree (MU/°)	0.40	0.75	0.75	0.20
Maximum MU per gantry degree (MU/°)	20	20	20	59
Minimum MU per arc segment (MU/seg)	0.1	1.0	1.0	0.2
Max leaf out of carriage distance (cm)	15	15	15	N/A
Maximum leaf tip difference (cm)	15	15	15	28

**Table 3:** Dose parameters and complexity metrics of the baseline plans for each machine. CL: contralateral, SC: spinal cord, BS: brainstem.

Parameter	CM	TBM	TBHD	VHD	VHD secondary
PTV1 D_95_ (Gy)	61.8	62.2	62.3	62.0	61.7
PTV1 D_50_ (Gy)	65.0	65.1	65.3	65.1	65.4
PTV1 D_0.03cc_ (Gy)	67.5	69.0	68.2	67.2	68.9
PTV2 D_95_ (Gy)	50.9	51.8	51.9	52.0	51.5
PTV2 D_50_ (Gy)	54.1	54.2	54.2	54.0	54.1
PTV2 D_0.03cc_ (Gy)	67.0	67.0	66.8	66.6	68.1
BS D_0.03cc_ (Gy)	37.6	36.0	35.5	34.2	37.7
SC D_0.03cc_ (Gy)	39.9	41.6	41.0	39.3	43.0
CL parotid mean (Gy)	21.3	24.0	24.2	23.1	21.4
Median MLC gap (mm)	39.6	38.1	38.5	16.9	18.3
SAS10	0.10	0.10	0.11	0.33	0.04
Mean MLC speed (cm/s)	0.7	0.7	0.7	1.4	1.5
Plan irregularity	7.5	7.9	10.0	14.3	15.0
Plan modulation	0.7	0.7	0.8	0.9	0.8
MCS as calculated with 0.5 mm minimum leaf gap	0.3	0.3	0.3	0.2	0.2
Mean gantry speed (°/s)	4.2	3.9	3.9	4.0	4.3
Gantry speed modulation	3.5	2.4	2.8	5.4	18.7

**Fig. 1:**
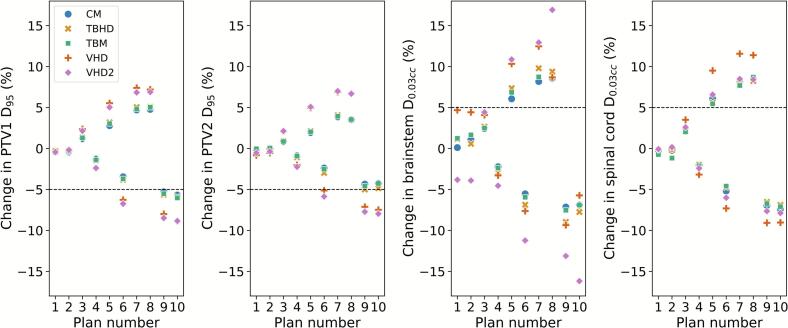
Change in DVH parameters for the modified plans relative to baseline plans computed with the same beam model for four linear accelerators: Varian Clinac (CM), TrueBeam HD (TBHD), TrueBeam Millenium (TBM), Elekta Versa HD (VHD) and a secondary VHD model (VHD2). Shown are the percentage changes in DVH parameters PTV1 D_95_, PTV2 D_95_, brainstem D_0.03cc_ and spinal cord D_0.03cc_.

Of the ten modified plans outlined in Table 1, in five the introduced errors were found to have a clinically significant dosimetric impact on either target coverage or dose to organs at risk (OARs) for the median model for all machines (Fig. 1, Table 4). Plan number six had a significant decrease in target coverage for the standard VHD and secondary models only. Similar trends across the machine models were also observed for other DVH parameters (Fig. S1, supplementary material).

**Table 4:** Any clinically significant dosimetric impacts of introduced plan modifications.

Plan	Dose metric	Mean change ± standard deviation (%) (‘standard’ models only)	Mean change ± standard deviation (%) (with VHD secondary)	Clinically significant impact?
1	BS D_0.03cc_	1.8 ± 1.7	0.7 ± 2.7	No
	PTV1 D_95_	−0.4 ± 0.1	−0.4 ± 0.1	No
2	BS _D0.03cc_	1.9 ± 1.5	0.8 ± 2.7	No
	PTV1 D_95_	−0.3 ± 0.1	−0.3 ± 0.1	No
3	BS D_0.03cc_	2.9 ± 0.7	3.2 ± 0.9	No
	PTV1 D_95_	1.5 ± 0.5	1.7 ± 0.5	No
4	BS D_0.03cc_	−2.7 ± 0.4	−3.1 ± 0.8	No
	PTV1 D_95_	−1.6 ± 0.4	−1.8 ± 0.5	No
5	BS D_0.03cc_	7.7 ± 1.6	8.3 ± 1.9	Yes
	PTV1 D_95_	3.6 ± 1.1	3.9 ± 1.1	No
6	BS D_0.03cc_	−6.5 ± 0.8	−7.4 ± 2.0	No
	PTV1 D_95_	−4.3 ± 1.1*	−4.8 ± 1.4	No
7	BS D_0.03cc_	9.8 ± 1.7	10.4 ± 1.9	Yes
	PTV1 D_95_	5.5 ± 1.1	5.8 ± 1.1	No
8	BS D_0.03cc_	8.8 ± 0.4	10.4 ± 3.3	Yes
	PTV1 D_95_	5.5 ± 1.0	5.8 ± 1.0	No
9	BS D_0.03cc_	−8.2 ± 0.9	−9.2 ± 2.1	No
	PTV1 D_95_	−6.1 ± 1.1	−6.6 ± 1.4	Yes
10	BS D_0.03cc_	−6.8 ± 0.7	−8.7 ± 3.8	No
	PTV1 D_95_	−6.6 ± 1.3	−7.1 ± 1.5	Yes

*Change in PTV1 D_95_ was notably larger for the VHD (−6.3 %) compared to other machines.

**Fig. 2:**
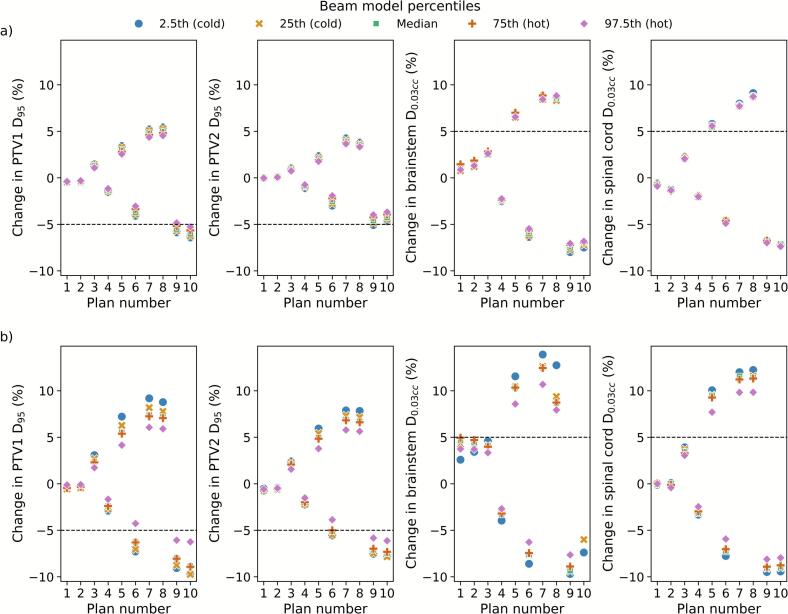
Change in DVH parameters for the modified plans relative to baseline plans for each beam model for: TBM (a) and VHD (b). Shown are the percentage changes in DVH parameters PTV1 D_95_, PTV2 D_95_, brainstem D_0.03cc_ and spinal cord D_0.03cc_. Percentile refers to which percentile of beam modelling parameters in Glen et al. [6] were used for the respective model.

Assessing the impact of variation in beam model parameters, a delivery error robustness evaluation for each beam model is plotted in Fig. 2. There was little variation (< ±1 %) in change in DVH parameters for the 2.5th and 97.5th percentile models for the TBM. However, larger variations were observed for the VHD models. The 97.5th percentile (hotter) beam models were found to be more robust with lower percentage differences from the baseline plans for both increases and decreases.

For the current plan dataset, the beam model variation did not impact whether simulated delivery errors for a plan were determined to have a clinically significant dosimetric impact for most cases. However, plan number nine for the TBM gave a clinically relevant (>5 %) PTV1 D_95_ underdosage for all beam models except the 97.5th percentile model which was at 5 %. Similarly for plan number six for the VHD, all beam models except for the 97.5th percentile model showed a >5 % PTV1 D_95_ reduction.

## 4 Discussion

A centralised planning approach was developed by refining a previously proposed methodology of assessing the sensitivity of participants’ PSQA systems via plans with purposely introduced errors. The central approach ensures that participants receive plans of similar quality meeting all clinical goals and, importantly, of similar robustness towards the introduced errors, as indicated by the small standard deviation across machine models in change in dose metrics in response to plan modifications. This resulted in similar dosimetric differences of the error plans from the original plan and thereby similar clinical relevance of the ability of a PSQA system or auditing system to identify plans correctly.

With making error plans the goal would generally be to have a number of plans each that should pass and fail PSQA. Relevant DVH parameters from the recalculated modified plans, as also shown here with D_95_ (PTV) and D_0.03cc_ (OAR), would be provided to the centre to support their evaluation process leaving it up to them to decide what should pass/fail. An exception would be if sensitivity and specificity were to be calculated by the study team. In this case, each plan must be classified as either “should pass” or “should fail”. This binary classification may not be ideal as whether a plan should pass or fail will often consider multiple factors. In such situations it is beneficial to have modifications that create dose changes which can be easily agreed upon whether they are acceptable or not, as also discussed below.

When introducing ten sample plan modifications, all machines presented a similar robustness with a standard deviation for change in brainstem D_0.03cc_ and PTV1 coverage of ≤1.7 % for all modified plans for the ‘standard’ models (excluding the VHD secondary model), i.e. similar variations of clinically significant dose parameters observed across the modified plans for each machine (Table 4). For this presented case, the VHD plans showed a slightly lower robustness to the implemented modifications (Fig. 1). However, the only case where dosimetric change was deemed significant (>5 % reduction PTV D_95_ or >5 % increase in OAR dose) for the VHD and not other machines is Plan 6. Hence this modification would likely be less useful for an audit. In order to cater for all departments including those with outlying machine limits the secondary VHD model would be necessary for this dataset. When including plans for this model, more variation in plan robustness was observed with standard deviation in change in dose metrics of up to 3.8 %. Therefore, ideally the use of models with outlying machine limits should be avoided if possible. The plan complexity metrics included in Table 3 showed a greater complexity for the baseline VHD plans compared to plans for other machines. Previous publications have expressed that plan complexity is an important consideration as part of plan quality and robustness and may influence the response of the plans to the introduced modifications [10,11]. However, the difference in robustness for both the standard VHD plan and the secondary VHD plan compared to the other machines was quite small and, through the proposed methodology, appropriate plan modifications where the variance is inconsequential can be identified. It should be noted that this lower robustness and higher complexity was observed for this specific case and may not be representative for the VHD in other cases.

Due to small variations in installation and beam tuning of individual linacs there will exist some level of beam model variation between facilities even for the same linac type [12,13]. These changes can affect the calculated dosimetric distribution which may also impact the robustness of plans. It is useful to estimate the magnitude of this impact for the intended PSQA assessment study prior to conducting, as was done here using published beam model data. Additionally, one can request re-calculations of the error plans from each centre to confirm validity of the approach. This is best to be done after PSQA results have been submitted to reduce the chance of bias.

For the here chosen situation, beam model variation had a greater effect on changes in DVH parameters for the VHD compared to other machines (Fig. 2). However, it should be noted the greater variation in beam modelling parameter data for this machine compared to others [6]. Previously, Hernandez et al. [14] investigated the difficulties of modelling the Elekta Agility MLC. Again, large differences in MLC modelling parameters were found between examined facilities which were attributed to diverse MLC configuration techniques. Further, it was concluded that the geometric characteristics of this MLC are not well modelled by current TPS parameters and therefore require compromises of available parameters.

For the presented head and neck case and introduced plan modifications, the impact of beam modelling parameters had minimal effect on whether modifications resulted in clinically significant changes for most plans. Care would need to be taken when analysing collected PSQA results for Plan 6 for the VHD and Plan 9 for the TBM, as the 97.5th percentile models produced DVH results which may be considered clinically acceptable while other beam models did not. It has previously been shown that inter-machine variability is small with relatively similar measured dose-calibration curves [13,14]. However, larger beam modelling differences may not necessarily result in inferior dose calculation as some parameters often compensate for others [12,14]. Conversely, the beam models presented in this work were intentionally designed so that beam modelling variations would be additive in effect, hence even extreme models possessed by participating facilities would be unlikely to approach the 2.5th and 97.5th percentile models here.

Despite the previously discussed benefits of centralised planning including reduced workload for participants, ease of scalability and the ability to benchmark participant results against each other, there are some limitations of this methodology. Centrally created plans may not represent clinically used planning characteristics for participating centres. However, in the case of PSQA audits, such as in the SEAFARER (sensitivity of patient specific quality assurance) study [3], the focus of the study was the performance of the PSQA system in detecting a clinically significant dosimetric difference as a result of introduced modifications. Therefore, the details of the plan in terms of being a match for the local clinic are less important.

One of the limitations of this study was the availability of beam modelling data for the TBHD using the RayStation TPS. IROC auditing data for Varian Eclipse and Phillips Pinnacle beam modelling parameters had similar magnitude of inter-centre deviation for the TBM and TBHD [6]. Therefore, the TBHD would likely demonstrate similar results with beam model variability to the TBM. As participant survey data continues to be collected, there may be the ability to better predict the range of parameter values necessary to encompass the community for TBHD. Continued collection of beam model data is necessary as this methodology relies on up-to-date data and fast collection of new data when substantial system upgrades are introduced.

The landscape of radiotherapy is consistently evolving, with the numbers of plans measured by PSQA gradually being reduced as confidence in treatment systems grows [[15], [16], [17]]. Currently, the majority of centres still perform PSQA regularly, however, this trend of reduced frequency is expected to continue. In this case, PSQA measurements will remain the ultimate back up to other methods and therefore it is even more important that the limited number of PSQA measurements completed have sufficient accuracy to confidently detect errors.

This work demonstrated how a centralized planning approach can be used to improve interpretability of results and inter-centre comparability of audit performance, with the example of remote PSQA assessment. Several challenges were addressed including developing dosimetrically similar plans across machines, accommodating varying machine limits and considering the impact of beam modelling differences. With this approach a relatively uniform robustness across linac types was achieved as well as an improved ability to include additional participants with minimal additional effort. This work lays the foundation for future assessment of centres in the context of remote PSQA assessment as well as other audit methodologies.

## CRediT authorship contribution statement

**José Antonio Baeza-Ortega:** Methodology, Software, Validation, Formal analysis, Investigation, Writing – original draft. **Lauren May:** Methodology, Software, Validation, Formal analysis, Investigation, Writing – original draft. **Mohammad Hussein:** Methodology, Resources. **Sarah Porter:** Data curation, Project administration. **Alisha Moore:** Data curation, Project administration. **Peter B. Greer:** Conceptualization, Methodology, Writing – review & editing, Supervision, Funding acquisition. **Catharine H. Clark:** Conceptualization, Methodology, Writing – review & editing, Supervision. **Joerg Lehmann:** Conceptualization, Methodology, Writing – review & editing, Supervision, Funding acquisition.

## Declaration of competing interest

The authors declare the following financial interests/personal relationships which may be considered as potential competing interests: JL is a consultant and auditor for the Australian Clinical Dosimetry Service (ACDS), the consulting QA physicist for the Trans-Tasman Radiation Oncology Group (TROG) and a Medical Physicist Advisor with SeeTreat PTY Ltd.
