# Supplementary material for: A proof of concept for improving comparability of dosimetry audits through centralised planning
Source: Phys Imaging Radiat Oncol. 2025 Nov 29;36:100879. doi: 10.1016/j.phro.2025.100879 (PMC12732305; doi:10.1016/j.phro.2025.100879)
Supplement: Supplementary Data 1 [file mmc1.pdf]

Table S1. Optimisation objectives used to create the mother plan and baseline treatment plans.

Function	ROI	Dose	Weight
Uniform dose	Plan PTV1	65 Gy	120
Uniform dose	Pseudo PTV2	54 Gy	100
Max dose	Spinal cord + 3 mm	44 Gy	100
Dose fall-off	Body (low dose distance 0.5 cm)	65 Gy (high), 40 Gy (low)	50
Dose fall-off	Body (low dose distance 1.5 cm)	65 Gy (high), 38 Gy (low)	30
Dose fall-off	Body (low dose distance 2.0 cm)	65 Gy (high), 20 Gy (low)	20
Max DVH	PseudoPTV2	57 Gy to 1% volume	10
Max EUD	IL_parotid	40 Gy (parameter A1)	10
Max EUD	CL_parotid	24 Gy (parameter A1)	10

Table S2. Clinical goals based on trial-specific quality assurance guidelines [7].

ROI	Parameter	Goal
PlanPTV1	D_{99}	> 90 % of radical dose (65 Gy)
PlanPTV1	D_{95}	> 95 % of radical dose (65 Gy)
PlanPTV1	D_{50}	100 % of radical dose (65 Gy)
PlanPTV1	D_{5}	< 105 % of radical dose (65 Gy)
PlanPTV1	D_{2}	< 107 % of radical dose (65 Gy)
PlanPTV2	D_{99}	> 90 % of elective dose (54 Gy)
PlanPTV2	D_{95}	> 95 % of elective dose (54 Gy)
PlanPTV2	D_{50}	100 % of elective dose (54 Gy)
Spinal cord	D_{max}	< 48 Gy
Brainstem	D_{max}	< 55 Gy
Spinal cord	V_{46Gy}	< 1cm^3
Spinal cord PRV	V_{48Gy}	< 1cm^3
Brainstem	V_{54Gy}	< 1cm^3
Brainstem PRV	V_{55Gy}	< 1cm^3
Contralateral parotid	Mean	< 24 Gy

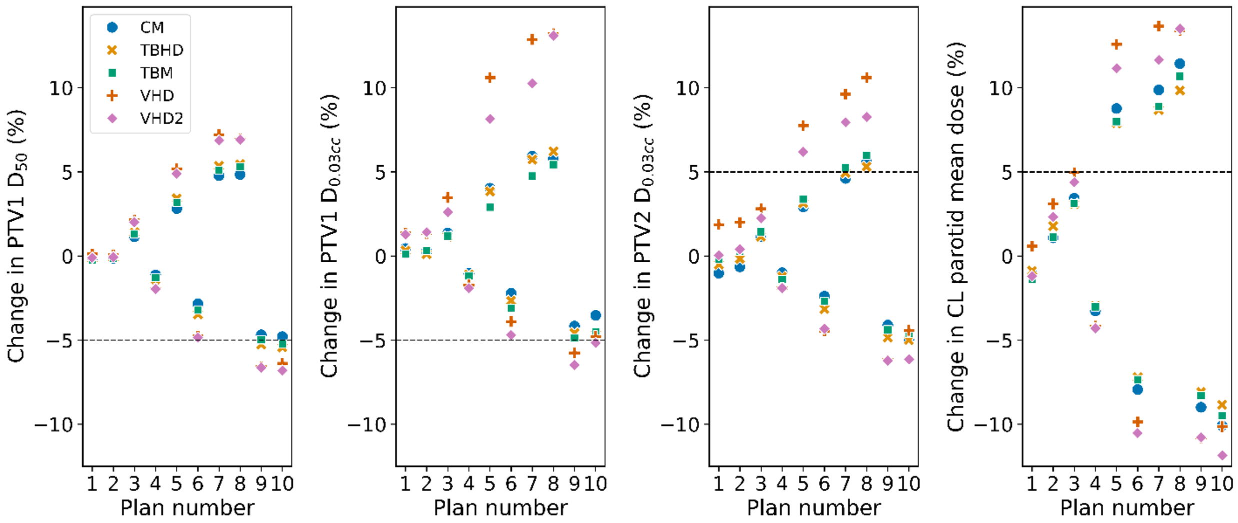

Figure S1. Change in DVH parameters for the modified plans relative to baseline plans computed with the same beam model for four linear accelerators: Varian Clinac (CM), TrueBeam HD (TBHD), TrueBeam Millenium (TBM), Elekta Versa HD (VHD) and secondary VHD model (VHD2). Shown are the percentage changes in DVH parameters PTV1 D_{50}, PTV1 $D_{0.03cc}$, PTV2 $D_{0.03cc}$ and contralateral (CL) parotid mean dose.
